# Spatial protein profiling reveals active roles for astrocytes in the chronic active lesion core during multiple sclerosis

**DOI:** 10.1007/s00401-025-02953-9

**Published:** 2025-11-01

**Authors:** Brandon C. Smith, Anthony Chomyk, Maria L. Habean, Benjamin C. Shaw, Rachel A. Tinkey, Bruce D. Trapp, Jessica L. Williams

**Affiliations:** 1 Department of Neurosciences, Cleveland Clinic Research, Cleveland Clinic, 9500 Euclid Avenue/NC30, Cleveland, OH 44195 USA; 2 Department of Neurosciences, Case Western Reserve University, Cleveland, OH 44016 USA; 3 School of Biomedical Sciences, Kent State University, Kent, OH 44240 USA

## Abstract

Chronic active lesions are a manifestation of multiple sclerosis (MS) and have been associated with disease progression. While astrocytes are heavily implicated in MS, little is known about their role in lesions, particularly in the lesion core. Here, we sought to gain insight into the spatial relationship between astrocytes and defined regions of chronic active lesions, and to better understand the environment within the relatively understudied lesion core, an area primarily composed of astrocytes. We analyzed four defined protein panels, focusing on astrocytes, in postmortem fresh-frozen cortical white matter tissue using NanoString GeoMx spatial protein profiling to compare normal appearing white matter (NAWM), the chronic active perilesion, rim, and core. We then performed immunofluorescent microscopy to determine the localization patterns of identified proteins within astrocytes. The most significant differences were observed between the chronic active lesion core and both NAWM and the perilesion. Proteins upregulated in the core relative to NAWM or the perilesion included the MAPK signaling pathway, immune checkpoint proteins, and indicators of phagocytosis. Our data indicate that astrocytes in the lesion core are distinct and actively influence the microenvironment. We posit that the differentially upregulated astrocytic signaling pathways, namely MAPK, immune checkpoints, and debris engulfment, are indicative of reactive astrocytes providing support to demyelinated axons by tempering the inflammatory milieu and clearing debris within the lesion core.

**Supplementary Information:**

The online version contains supplementary material available at 10.1007/s00401-025-02953-9.

## Introduction

Multiple sclerosis (MS) is a chronic inflammatory and demyelinating disease of the central nervous system (CNS) that leads to progressive neurological disability [1]. During MS, demyelination disrupts axon signal conduction, which leads to a wide range of symptoms including muscle weakness, sensory disturbances, vision complications, and cognitive decline [1, 2]. MS pathophysiology encompasses both inflammation and neurodegeneration, which manifests as CNS lesions and a reduction in total brain volume [3, 4]. Initially, lesions are primary points of damage and include infiltrating and activated immune cells, damage to myelin, and axonal injury [1, 2, 4]. In chronic active white matter lesions, microglia and macrophages form a distinct rim around a gliotic lesion core, which represents an area of prolonged damage lacking myelin [5–7].

Imaging of chronic active white matter lesions has suggested that lesion expansion is associated with augmented neurological disability in progressive MS and is likely indicative of ongoing demyelination [7]. Axonal transection is less prevalent in the chronic active lesion core compared to the lesion edge [2], where demyelinated axons reside, suggesting that the core may be a more quiescent environment. Continued preservation of demyelinated axons within the lesion core could slow disease progression; however, compared to the lesion rim, the core of chronic active lesions is vastly understudied. Gaining insight into the ongoing cellular processes active within the lesion core may yield novel mechanisms to protect the CNS from further damage and potentially alter MS disease course. While current disease-modifying therapies have proven beneficial for inflammatory stages of MS, these treatments are largely immunomodulatory and have had little impact on progressive neurodegeneration [8].

Astrocyte activation is a hallmark of MS throughout disease and their altered morphology in and around lesions suggests a substantial change in function [9]. Astrocytes were initially seen as pathologic bystanders during MS, but are becoming increasingly appreciated as active participants in the disease process [10]. Astrocytes are the primary cells forming the gliotic scar in chronic active and inactive lesions, and they can assume both protective and detrimental roles during disease progression, depending upon the complex signals that they receive [11–15]. This can lead to astrocytes contributing to ongoing demyelination and axonal damage, promoting repair and protecting axons from further damage, or even both simultaneously [15–17].

While astrocytes comprise the vast majority of cells present in the chronic active lesion core [9, 18], the functional significance of their altered morphology remains largely unknown. We examined the protein profiles of astrocytes in four distinct regions in and around chronic active lesions to advance our understanding of lesion pathophysiology. Our findings support the concept that astrocytes are an active participant in modulating the lesion environment during MS.

## Materials and methods

### Human postmortem tissue

All postmortem human tissue used was collected as part of the tissue procurement program approved by the Cleveland Clinic Institutional Review Board. Tissue donation from patients with MS was obtained with consent from the patient or next of kin. Tissue procurement and processing of MS, amyotrophic lateral sclerosis (ALS), and control tissues at the Cleveland Clinic have been described in detail [19]. MS subtypes and demographics of the patient cohort used for these studies are listed in Table 1. Briefly, brain tissue was collected and sliced (1 cm thick) using a guided box. Slices were either rapidly frozen for biochemical analysis or short fixed in 4% paraformaldehyde (PFA), followed by sectioning for morphological studies. Demyelination of white matter lesions was confirmed by proteolipid protein (PLP) immunostaining and chronic active lesions were further characterized by staining adjacent sections for major histocompatibility complex class II (MHCII) and visualizing the microglia/macrophages that accumulate at the lesion border, as described previously [20].

**Table 1:** Patient demographics

Patient	MS type	Donate age	Sex	Disease duration (years)	Final EDSS
MS 1	SPMS	53	M	15	9.5
MS 2	SPMS	61	F	35.3	9.5
MS 3	SPMS	45	M	36	7
MS 4	PPMS	57	F	15.2	6.5
MS 5	SPMS	48	F	27.1	9
MS 6	SPMS	56	M	32.7	9.5
MS 7	SPMS	60	F	29.5	9
MS 8	SPMS	59	F	37.5	9
MS 9	PPMS	51	F	14.9	7.5
MS 10	SPMS	59	F	22.9	9.5
MS 11	SPMS	40	F	19.8	9.5
MS 12	SPMS	35	M	21	9.5
MS 13	RRMS	48	F	N/A	N/A
MS 14	PPMS	52	F	29	8
MS 15	SPMS	68	F	33	8.5
MS 16	SPMS	70	M	41	8.5
MS 17	PPMS	55	F	36	9
Control 1	N/A	34	F	N/A	N/A
Control 2	N/A	96	F	N/A	N/A
ALS 1	N/A	56	M	N/A	N/A
ALS 2	N/A	59	F	N/A	N/A

Patients listed were used for spatial protein profiling and/or immunohistochemical analysis

*EDSS* Expanded Disability Status Scale, *SPMS* secondary progressive MS, *PPMS* primary progressive MS

### Digital spatial profiling

Digital spatial profiling was performed using the GeoMx® Digital Spatial Profiler instrument (NanoString) following the manufacturer’s protocols. Briefly, fresh-frozen periventricular white matter from patients with MS (Table 1) containing identified chronic active lesions was sectioned at a thickness of 10 µm. Slides were baked at 65 °C for 15 min and fixed with cold 95% ethanol. Slides were blocked with Buffer W (NanoString) for 2 h and stained using a rabbit anti-ALDH1L1 antibody, which was conjugated to Alexa Fluor^®^ 647 (Cell Signaling, 85828S). Following a 30 min post-fix with 4% PFA, slides were labeled with the nuclear marker SYTO13 (NanoString). Individual regions of interest (ROIs) were drawn using adjacent chronic active lesion tissue sections labeled for PLP and MHCII. ROIs included normal appearing white matter (NAWM), perilesional white matter, lesion rim, and lesion core.

All slides were profiled using 56 specified targets from four panels (Neural Cell Subtyping Panel, Glial Cell Subtyping Panel, IO Drug Target Panel, and MAPK Signaling Panel), which included three housekeeping proteins (GADPH, histone H3, and ribosomal protein S6) and three negative controls (mouse IgG1, mouse IgG2a, and rabbit IgG) (Fig. 2). Protein targets were identified by bound photocleavable oligonucleotide-conjugated antibodies (NanoString). Cleaved oligonucleotides were hybridized at 37 °C for 16–24 h. Barcodes from each ROI were quantified using the nCounter® platform (NanoString). Read counts were normalized against nCounter®-specific internal controls and then each individual ROI was normalized to the average of GAPDH and histone H3 read counts. Ribosomal protein S6 was not included as a housekeeping protein, as its expression level was altered by region.

### Immunohistochemistry

To validate the cellular specificity and location of differentially regulated proteins, immunohistochemistry was performed as previously described [20]. Briefly, lesions identified in human periventricular white matter (Table 1) were subjected to antigen retrieval by briefly boiling free-floating sections in 10 μM citrate buffer. Sections were blocked with 5% goat serum and 0.03% Triton X-100 (Sigma-Aldrich) for 1 h at room temperature and then exposed to antibodies specific for GFAP (Invitrogen, 13-0300), Iba1 (Wako, 019-19741), SMI31/32 (BioLegend, 801601/801701), ALDH1L1 (Cell Signaling, 85828S), EGFR (R&D Systems, AF231), p44/42 ERK1/2, (Cell Signaling, 4696T), phospho-p90RSK (Cell Signaling, 8753T), 4-1BB (ThermoFisher, 14-9056), Tim-3 (BioLegend, 119702), MERTK (R&D Systems, AF591), CTSD (Novus, MAB1029), TMEM119 (BioLegend, 853301), LAMP1 (Abcam, AB25630), and CD68 (Novus Biologicals, NBP2-33337) for 3–5 days at 4 °C. Sections were then washed and secondary antibodies conjugated to Alexa Fluor 405, 488, 555, or 647 (ThermoFisher Scientific) were applied for 1 h at room temperature. Sections were then treated with 0.3% Sudan black in 70% ethanol for 3 min. Finally, sections were imaged using a Zeiss LSM800 confocal microscope (Carl Zeiss) and z-stacks were reconstructed using Imaris imaging software.

### BioTuring

BioTuring Lens software was utilized for downstream bioinformatics analysis of target genes using the single-nucleus RNA sequencing dataset of white matter MS lesions from Absinta et al*.* [21]. Using the Talk2Data function, the dataset was uploaded and subclustered for astrocytes and further subclustered into different regions of chronic active lesions. The UMAP analysis function was used to identify and plot target genes.

### GWAS secondary analysis

Summary statistics from the International Multiple Sclerosis Genetics Consortium were used to identify variants in selected gene loci for significant associations with disease onset [22] or severity [23]. Locus zoom plots were built using the locuszoomr package version 0.3.8 in R 4.4.2 using a 50 kb flank of the selected gene identified using GRCh37 (per the original datasets).

### Statistical analysis

Data were analyzed using a one-way ANOVA with Tukey’s correction for multiple comparisons where appropriate. Data presented in volcano plots were analyzed using the two-stage Benjamini–Krieger–Yekutieli method for multiple comparisons to control the false discovery rate. All statistical analyses were performed using GraphPad Prism Version 10.1.2 software (GraphPad). A *p* value of less than 0.05 was considered statistically significant.

## Results

### Astrocyte process coverage, morphology, and interactions differ between regions of chronic active lesions

**Fig. 1:**
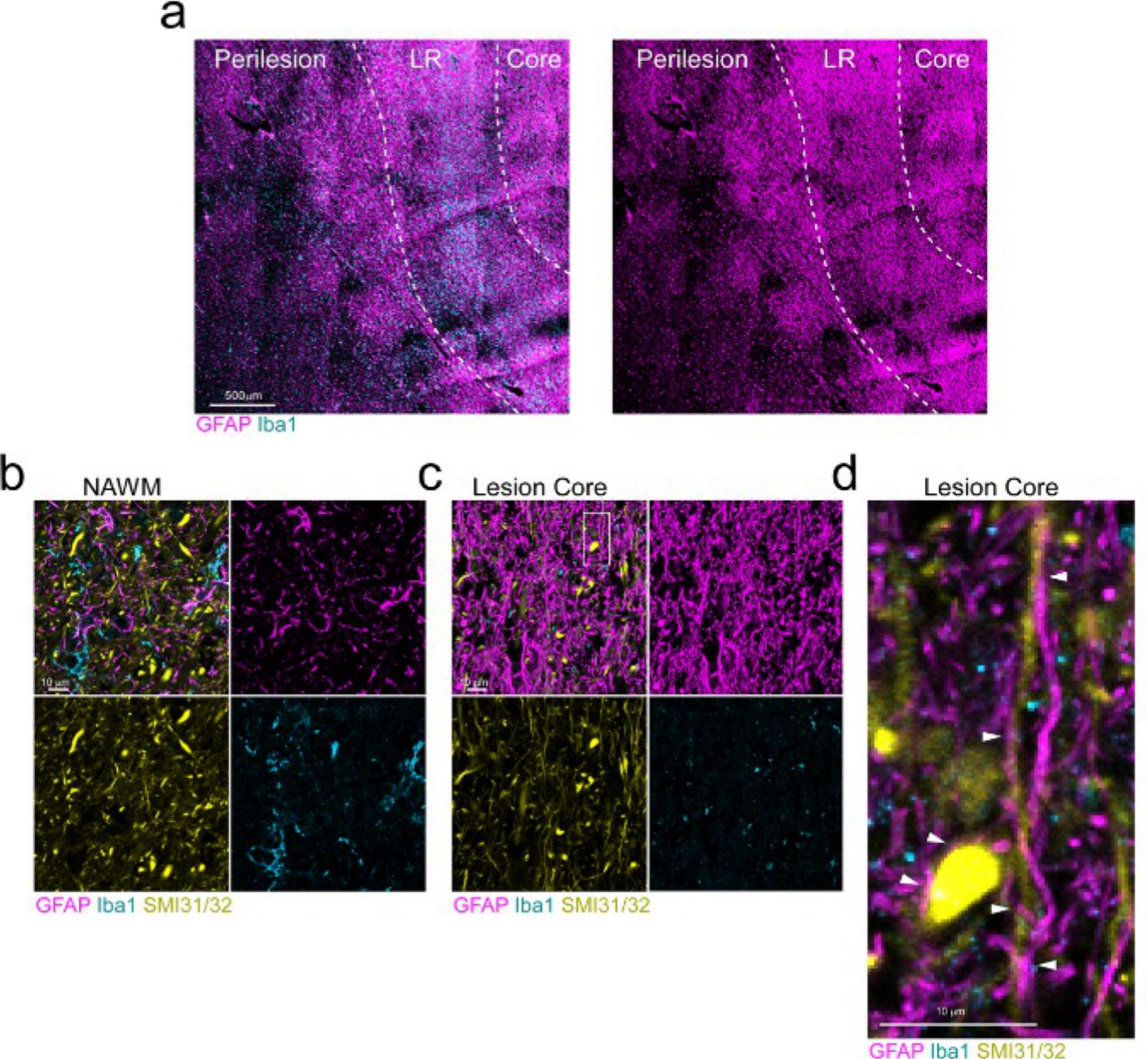
Astrocyte coverage, morphology, and cellular interactions differ spatially across chronic active lesions. Astrocytes, myeloid cells, and axons were identified using antibodies directed against GFAP, Iba1, and SMI31/32, respectively. **a** The density of Iba1^+^ cells was used to identify the lesion rim, outlined with white dotted lines. Astrocyte GFAP density was increased in the lesion rim and core compared to NAWM. **b** NAWM astrocytes appeared less reactive and more diffuse compared to **c** the lesion core, where **d** astrocytes maintained close association with axons, as indicated by white arrowheads. Scale bars, 500 μm (**a**) and 10 μm (**b–d**)

Immunofluorescent (IF) imaging using GFAP labeling showed that astrocyte morphology is greatly altered in and around chronic active lesions. Using Iba1 to label myeloid cells and identify the lesion rim, astrocyte density intensified moving through the perilesion and became increasingly gliotic approaching the lesion core (Fig. 1a). Higher magnification shows significant GFAP density changes within the lesion core compared to NAWM (Fig. 1b, c). Importantly, core-associated astrocyte processes were closely associated with remaining SMI-labeled axons, some of which appear to have swollen retraction bulbs (Fig. 1c, d). These data demonstrate significant morphological heterogeneity between astrocytes and their processes from the perilesion that continues into the core, indicating that the function of astrocytes may differ between regions of the chronic active lesion. Further, the close association of astrocytic processes with axons in the core suggests a vital interaction that could have either protective or detrimental consequences.

### Distinguishing the regional protein profiles of astrocytes in chronic active lesions

**Fig. 2:**
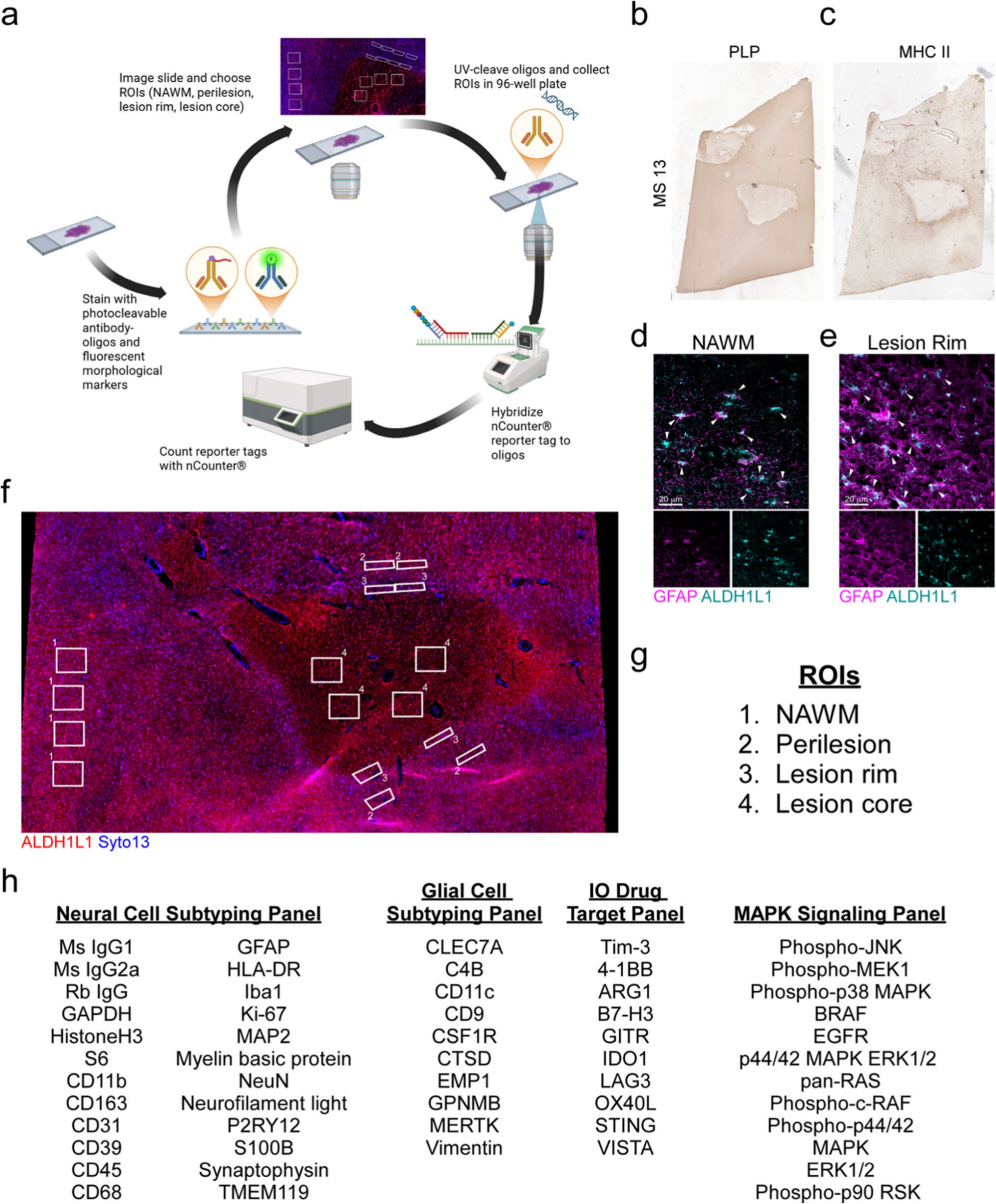
Parameters of the NanoString GeoMx® Digital Spatial platform. **a** Schematic representation of the NanoString GeoMx® Digital Spatial platform protocol. Chronic active lesions were characterized using **b** PLP to assess myelin loss and **c** MHCII to identify the lesion rim. ALDH1L1 was used for identification of astrocytes in both **d** the NAWM and **e** lesion and demonstrated co-localization with GFAP. **f** Regions of interest labeled with ALDH1L1 and SYTO13 were collected from **g** NAWM, the perilesion, lesion rim, and lesion core areas. **h** Spatial protein profiling analysis of collected ROIs was conducted using antibody panels for neural cell subtyping, glial cell subtyping, IO drug targets, and MAPK signaling. Schematic in **a** was created using BioRender.com

To gain insight into the functions of astrocytes in and around chronic active lesions, we utilized cell-specific spatial protein profiling to examine labeled astrocytes within defined ROIs. We used the GeoMx® Digital Spatial platform as outlined in Fig. 2a. ROIs were identified using tissue maps of characterized chronic active lesions labeled with PLP and MHCII in serial sections (Fig. 2b, c). Since GFAP labeling was ubiquitous throughout the lesion and closely associated with other cell types (Fig. 1), it presented as a poor morphological marker for isolating astrocytes as a spatial identifier. To specifically mask for astrocytes within designated ROIs, we employed the astrocyte-specific marker ALDH1L1, as it primarily labels the cell body and large processes and was relatively evenly distributed between ROIs (Fig. 2d, e). Following tissue processing, the lesions were scanned, and ROIs were identified based upon proximity to the MHCII^+^ microglial/macrophage line from serial sections and nuclear density (Fig. 2f, g). The protein panels assessed included those related to cell identification and activation status (Neural Cell Subtyping, Glial Cell Subtyping), confirmed and feasible drug targets (IO Drug Target), and those that included indicators of cell survival, proliferation, and inflammation (MAPK Signaling) (Fig. 2h). Together, these panels allowed for comparison of the activation and functional status of astrocytes between various regions of the chronic active lesion and provide insight into potential drug targets.

### Astrocytes from the lesion core are the most spatially diverse

**Fig. 3:**
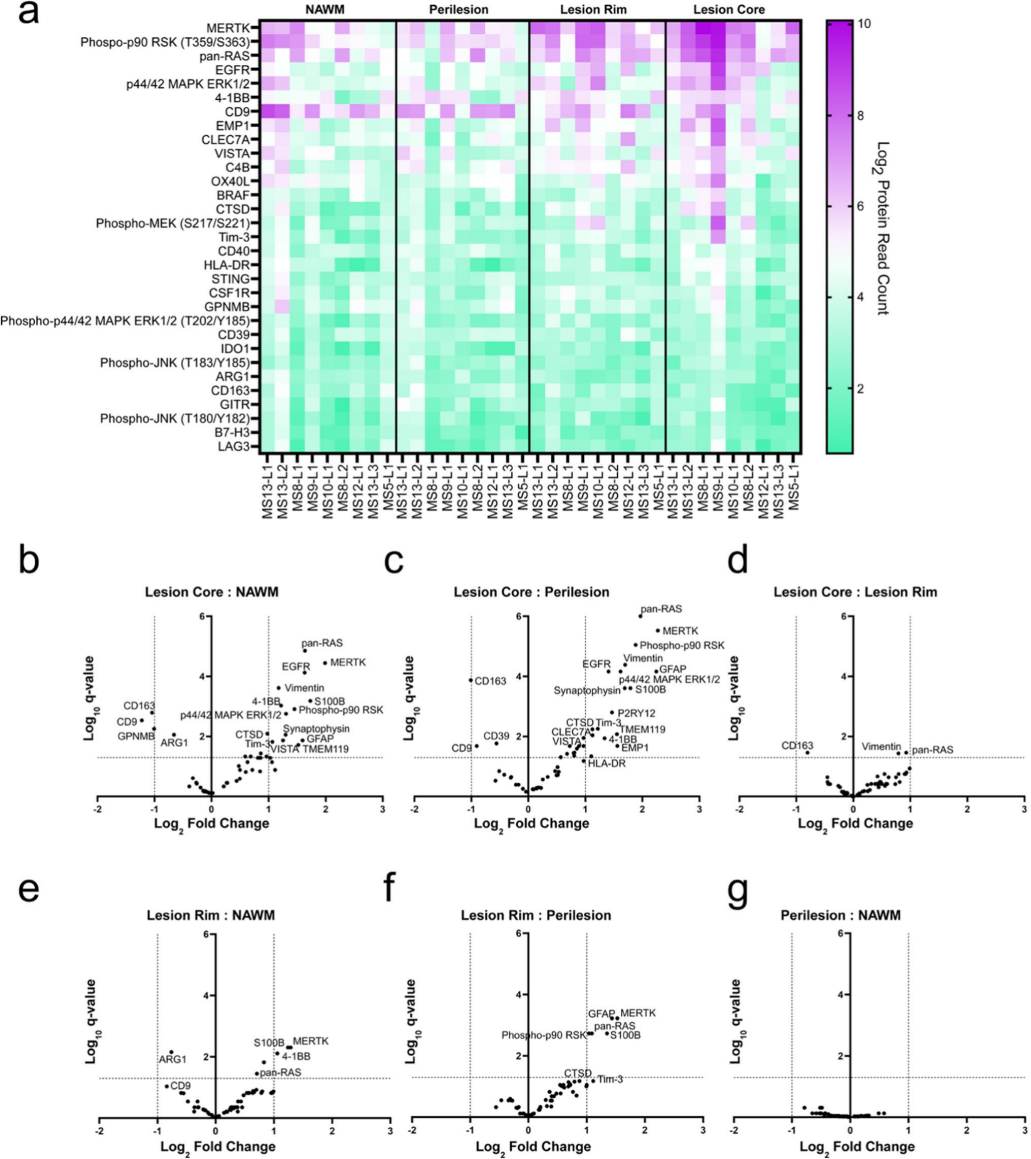
Lesion core astrocytes are spatially diverse. **a** Normalized protein read count was subdivided by ROI across patient lesions and values were sorted by lesion core expression. Astrocytes in the lesion core had the largest number of differentially expressed proteins compared to the **b** NAWM and **c** perilesion but were mostly similar to the **d** lesion rim. Modest changes in protein expression were seen in the lesion rim compared to the **e** NAWM and **f** perilesion, while no significant changes were observed in the **g** perilesion compared to the NAWM. Significant *q* values were set at > 1 or < − 1 and were determined using the Benjamini–Krieger–Yekutieli multiple comparison analysis for false discovery

Analysis of normalized protein levels per ROI revealed that astrocytes in the lesion core exhibited the most distinct protein expression profile compared to those in other regions (Fig. 3a). This trend was the most prominent comparing the lesion core to the NAWM and perilesion and diminished at the lesion rim (Fig. 3b–d). While astrocytes within the NAWM and perilesion were similar, the lesion rim had a modest amount of differentially expressed proteins compared to both the NAWM and the perilesion (Fig. 3e–g). These data are corroborated by single-nucleus RNA sequencing of chronic active lesions reported by Absinta et al*.* [21], where the largest astrocytic transcriptional differences similarly occurred in the lesion core (Supp. Figure 1a, b). These data reveal a distinct molecular signature of astrocytes in the chronic active lesion core, setting it apart from other regions, particularly the NAWM. Based on transcriptome analysis, it is known that the chronic active lesion contains a multitude of reactive glial cell phenotypes [21]; however, here we describe for the first time the partial protein profile of spatially distinct astrocytes to begin to assess their functional significance.

### EGFR signaling in lesion core-associated astrocytes

**Fig. 4:**
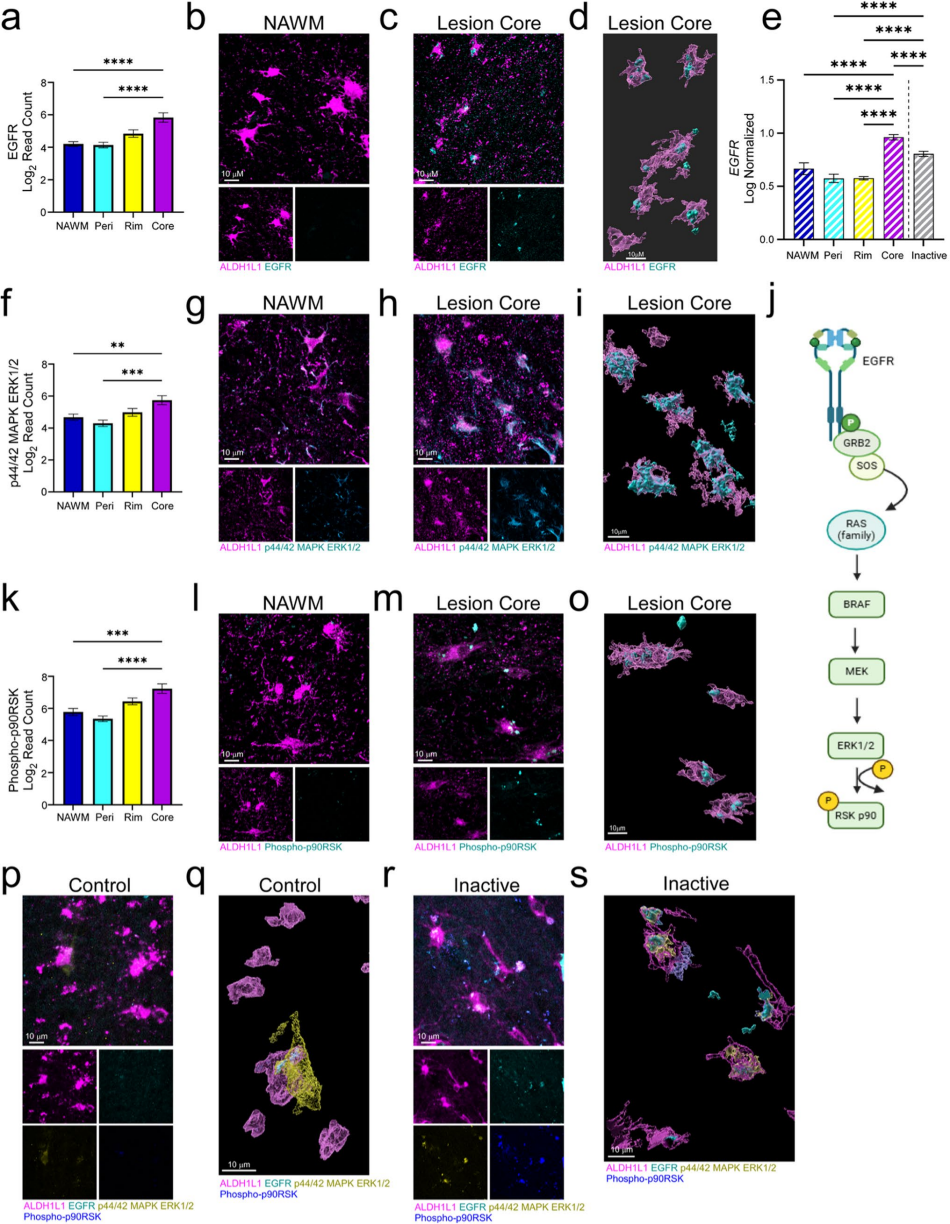
MAPK pathway proteins are upregulated in lesion core astrocytes. **a** Quantification of EGFR protein expression across ROIs revealed elevated expression in the lesion core compared to NAWM and the perilesion. **b, c** IF labeling and **d** high-resolution 3D reconstruction using Imaris software confirmed EGFR protein expression in lesion core astrocytes. **e** Reanalysis of transcriptomic data [21] using BioTuring Lens software revealed a corresponding increase in *EGFR* transcript within astrocytes in the core compared to other regions of chronic active lesions. **f** p44/42 MAPK ERK1/2 expression was similarly found to be elevated in the lesion core and confirmation of astrocyte-specific expression was demonstrated via **g, h** IF labeling and **i** Imaris 3D reconstruction. **k** Additionally, effector molecules of the MAPK pathway, including phospho-p90RSK, had a corresponding pattern of expression. **l, m** IF labeling confirmed increased expression in the lesion core compared to NAWM and **o** 3D reconstruction further demonstrated overlap with astrocytes. **j** Schematic representation of the EGFR pathway demonstrates the activation order of target proteins. **p** IF labeling and **q** high-resolution 3D reconstruction using Imaris software demonstrated the lack of EGFR, p44/42 MAPK ERK1/2, and phospho-p90RSK protein expression in control tissue astrocytes. **r** IF labeling and **s** Imaris 3D reconstruction confirmed EGFR, p44/42 MAPK ERK1/2, and phospho-p90RSK protein expression in chronic inactive lesion astrocytes. Differences between groups were tested using a one-way ANOVA with Tukey's test for multiple comparisons. Error bars = mean ± SEM. *p < 0.05, **p < 0.01, ***p < 0.001, ****p < 0.0001. Scale bars, 10 μm. Schematic in **j** was created using BioRender.com

Multiple proteins within the MAPK signaling pathway, including EGFR and p44/42 MAPK ERK1/2 (ERK1/2), were upregulated in the core relative to both the NAWM and perilesion, indicating potential activation of the EGFR signaling axis (Fig. 4a, f). EGFR signaling is critical for maintenance of astrocyte growth and differentiation and is also associated with axonal growth and guidance [24]. Additionally, subsequent protein kinase (ERK1/2) activation plays a role in many cellular processes including survival, proliferation, growth, and inflammation [25, 26]. Further, a downstream effector (Fig. 4j), phospho-p90RSK, was also increased (Fig. 4k) and of the proteins analyzed, is one of the only phosphorylated proteins upregulated in lesion core astrocytes. Phospho-p90RSK maintains a critical role in a multitude of cellular functions including, but not limited to, growth factor signaling and cellular metabolism that may vary depending on cell type [27].

Validation of EGFR-related proteins via IF staining showed increased expression of all three targets in the lesion core compared to NAWM (Fig. 4b, c, g, h, l, m; Supp. Figure 4a–f), and 3D reconstruction using Imaris software confirmed astrocyte-specific expression (Fig. 4d, i, o). To determine if any of these proteins followed similar transcriptional trends, comparative analysis was conducted using a publicly available spatial transcriptomic dataset [21]. Notably, *EGFR* was the only transcript found to be significantly increased in astrocytes in the core of chronic active lesions (Fig. 4e). However, additional GWAS analyses revealed significant associations between single nucleotide polymorphisms (SNPs) in the ERK1/2 genes, *MAPK1* and *MAPK3*, in both MS onset [22] and severity [23] (Supp. Figure 2b, c, e, f). To determine if this pattern of protein expression was unique to the chronic active lesion core, we performed IF labeling of control and ALS tissue as well as chronic inactive lesion core tissue. Of note, although ALS is traditionally considered a gray matter disease, demyelination and oligodendrocyte death have been observed [28]. Analysis of labeled tissues revealed that none of the EGFR signaling targets were expressed in control tissue astrocytes (Fig. 4p; Supp. Figure 4 g), only astrocytic ERK1/2 was observable in ALS tissue (Supp. Figure 3a, d), and all three were present in the chronic inactive lesion core (Fig. 4r; Supp. Figure 4 h). We used 3D reconstruction to confirm these expression profiles (Fig. 4q, s). These findings further support the importance of the EGFR and MAPK signaling pathways in MS lesion core astrocytes.

### Immune checkpoint proteins are expressed by lesion core astrocytes

**Fig. 5:**
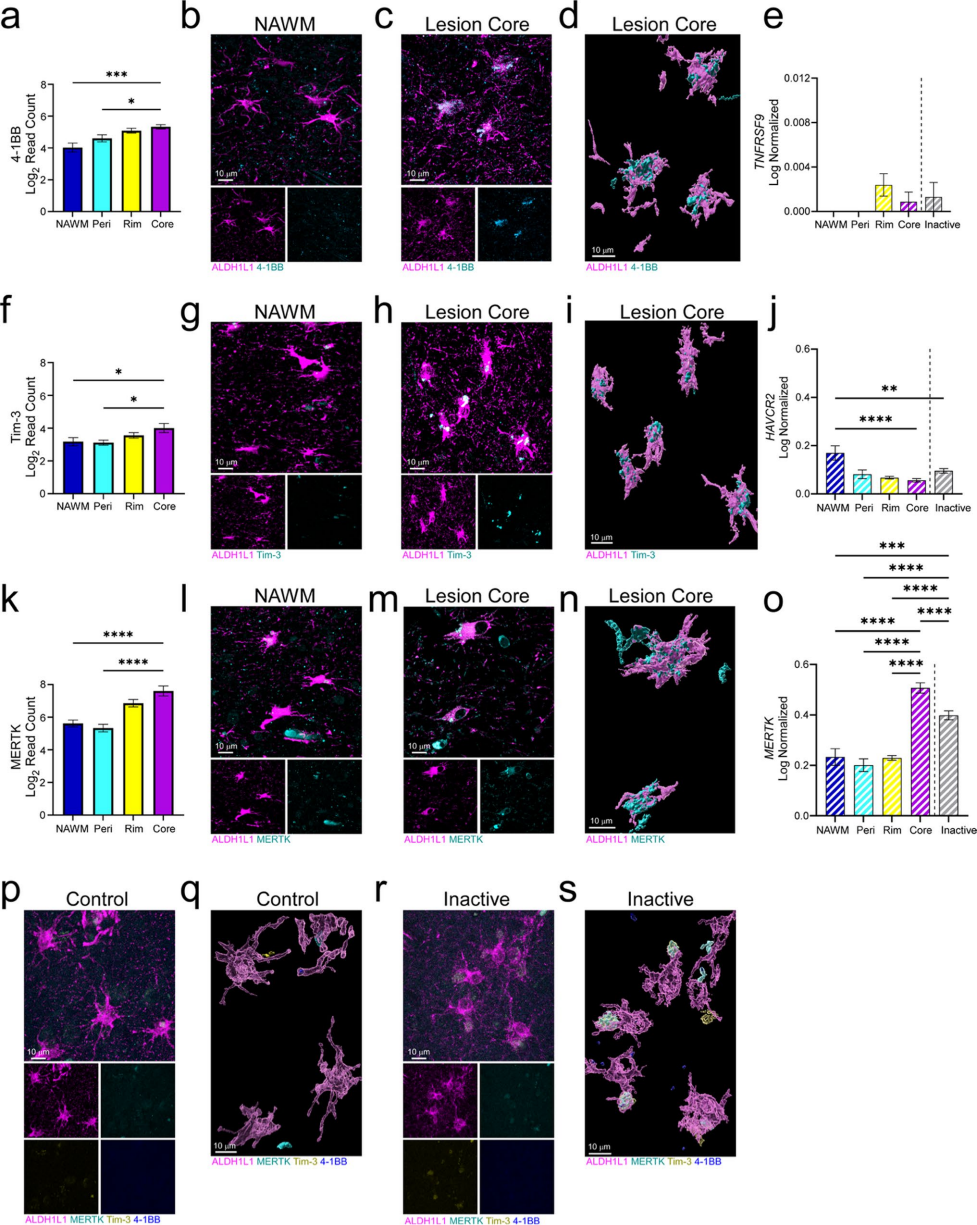
Immune checkpoint proteins are increased in lesion core-associated astrocytes. **a** Levels of 4-1BB protein were quantified across ROIs and demonstrated highest expression in the lesion core compared to NAWM and perilesion. **b, c** Imaging of 4-1BB co-localization with ALDH1L1 showed elevated protein expression within the lesion core compared to the NAWM and was further validated using **d** Imaris 3D reconstruction. **e** No significant changes in *TNFRSF9* (4-1BB) transcript were observed across ROIs. **f** Tim-3 followed a similar expression pattern across ROIs. **g, h** IF imaging demonstrated increased protein expression in lesion core astrocytes compared to NAWM, which was corroborated by **i** Imaris 3D reconstruction. **j** *HAVCR2* (Tim-3) transcript levels were highest in NAWM astrocytes compared to other ROIs. **k** Protein profiling analysis revealed that MERTK was most highly expressed in lesion core astrocytes compared to NAWM and perilesion. **l, m** Elevated MERTK expression was confirmed in the lesion core compared to NAWM via IF labeling and **n** 3D reconstruction further illustrated expression in astrocytes. **o** A corresponding increase in *MERTK* transcript within astrocytes was demonstrated in the lesion core compared to other areas. IF labeling combined with 3D Imaris reconstruction of **p, q** control tissue and **r, s** chronic inactive lesions confirmed the lack of observable MERTK, Tim-3, and 4-1BB protein expression in ALDH1L1^+^ astrocytes. Transcript data was sourced from Absinta et al*.* [21]. Differences between groups were determined using a one-way ANOVA with Tukey's test for multiple comparisons. Error bars = mean ± SEM. *p < 0.05, **p < 0.01, ***p < 0.001, ****p < 0.0001. Scale bars, 10 μm

Immune checkpoints are proteins of the immune system that modulate the duration and amplitude of an inflammatory response to maintain self-tolerance and minimize tissue damage [29]. We and others have recently described the influence of astrocytic immune checkpoint expression during neuroinflammation, and specifically in chronic active MS lesions [30, 31]. Similarly, here we report that lesion core astrocytes upregulate several proteins involved in the regulation of the immune response by immune checkpoints compared to NAWM and perilesion astrocytes including 4-1BB, Tim-3, and MERTK (Fig. 5a, f, k). 4-1BB, a co-stimulatory receptor and member of the TNFR superfamily, has primarily been studied in T cells and can vary in function depending on the inflammatory context and cell types involved [32, 33]. In murine obesity-induced inflammation, upregulation of astrocytic 4-1BB led to increased inflammation [34]; however, in a murine model of MS, agonism of 4-1BB inhibited autoreactive T cell responses and limited clinical relapse [35]. Tim-3 has been primarily studied in cancers but has been found to be upregulated by astrocytes during CNS injury and to reduce inflammation, acting as an inhibitory immune checkpoint [36, 37]. MERTK is commonly known to facilitate the phagocytosis of apoptotic cells [38], but it also stimulates the PD-1/PD-L1 axis [39], a prominent inhibitory checkpoint pathway known to be expressed in chronic active lesions [30].

We visualized these immune checkpoint-associated proteins using IF co-labeling with ALDH1L1 in both the NAWM and lesion core of chronic active lesions, validating that 4-1BB, Tim-3, and MERTK were expressed and localized with astrocytes in the lesion core (Fig. 5b, c, g, h, l, m; Supp. Figure 5a–f). 3D reconstructions of astrocytes in the lesion core were generated to highlight the prominence and cellular localization of these immune checkpoint proteins (Fig. 5d, i, n). Using spatial transcriptomics, Absinta et al*.* [21] found that while transcript levels of *TNFRSF9* (4-1BB) did not differ between regions, *MERTK* followed a similar expression profile to that of protein, where expression was most pronounced in core astrocytes (Fig. 5e, j). Interestingly, *HAVCR2* (Tim-3) transcript levels had the opposite trend compared to spatial protein expression, with *HAVCR2* being the highest in NAWM and diminishing approaching the lesion core (Fig. 5o). Of note, the immune checkpoint targets investigated were largely absent in astrocytes from control, chronic inactive MS lesions (Fig. 5p–s; Supp. Figure 5g, h), and ALS tissue (Supp. Figure 3b, e). These findings emphasize the power of combining spatial transcriptomics with protein analyses and suggest that astrocytes may work to modulate the immune profile of the lesion core microenvironment.

### Debris clearance in the lesion core

**Fig. 6:**
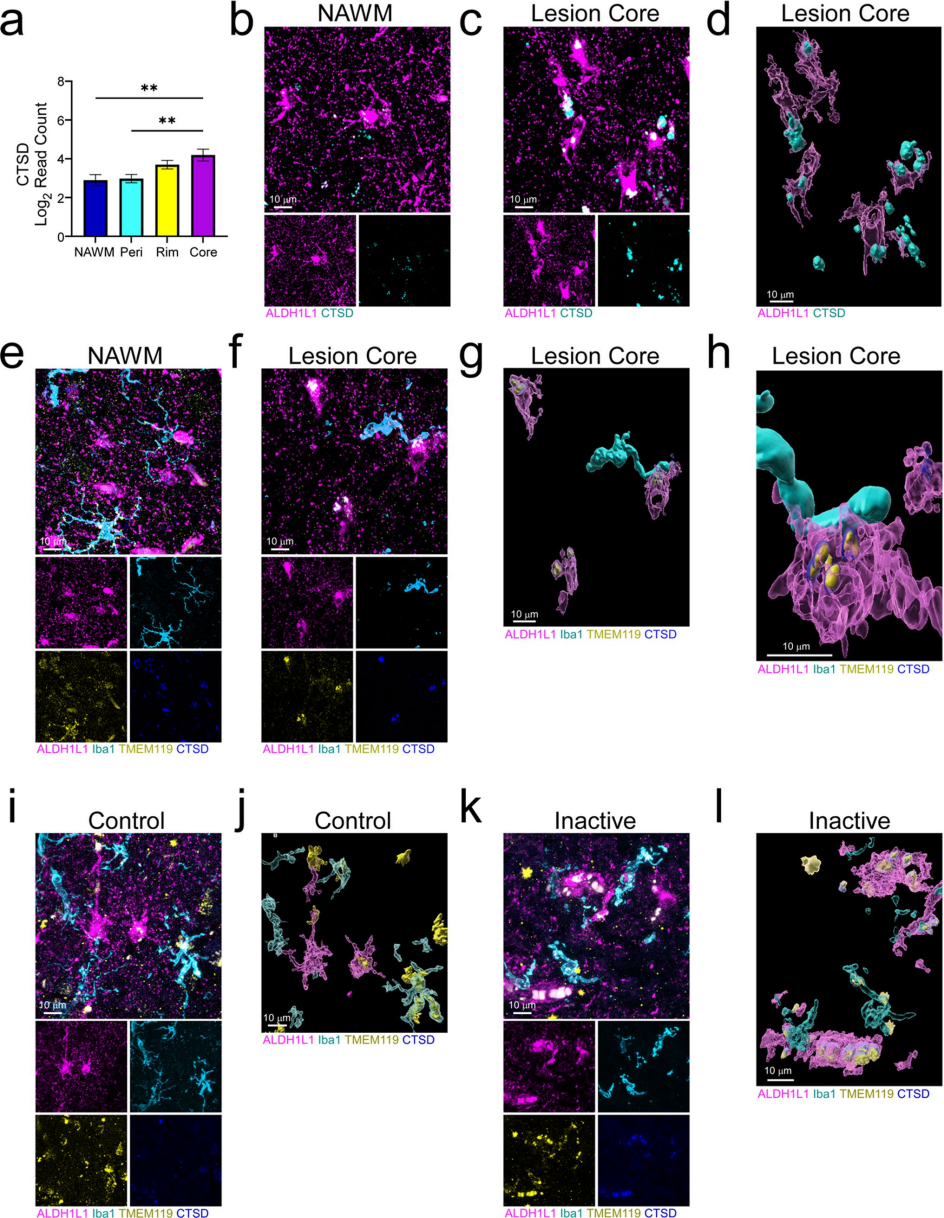
Protein expression profiles in lesion core astrocytes indicate that they are phagocytically active. **a** Spatial protein profiling of astrocytes in chronic active lesions demonstrated increased expression of CTSD in the lesion core compared to NAWM and perilesion. **b, c** Protein expression was validated via IF imaging and revealed increased expression in astrocytes in the lesion core compared to the NAWM. **d** 3D reconstruction using Imaris software confirmed expression within ALDH1L1^+^ astrocytes. **e, f** TMEM119 was expressed within astrocytes in the lesion core, but not in the NAWM, and **g, h** 3D reconstruction illustrated direct overlap of TMEM119 within CTSD^+^ lysosomes in lesion core astrocytes. **i** IF labeling and **j** high-resolution 3D reconstruction using Imaris software confirmed TMEM119 and CTSD protein expression in control tissue microglia with an apparent absence in astrocytes. **k** IF labeling combined with **l** high-resolution 3D reconstruction revealed TMEM119 and CTSD protein expression in chronic inactive lesion astrocytes. Differences between groups were tested using a one-way ANOVA with Tukey's test for multiple comparisons. Error bars = mean ± SEM. *p < 0.05, **p < 0.01, ***p < 0.001, ****p < 0.0001. Scale bars, 10 μm

While astrocytes are not classically thought of as highly phagocytic cells, this function of astrocytes has been highlighted more prominently in the last decade. Microglia and astrocytes have several phagocytosis receptors in common that are used for engulfment of dead and dying cells in the CNS, including MERTK [40]. Interestingly, spatial protein profiling analysis revealed astrocytic expression of another critical mediator of ingestion and degradation in the CNS, cathepsin D (CTSD). CTSD is a lysosomal protease critical for the autophagy-lysosomal system in the CNS [41] and was increased in lesion core astrocytes compared to those in NAWM and in the perilesion (Fig. 6a). We visualized the expression of CTSD in the context of ALDH1L1^+^ astrocytes and found that CTSD co-localization with astrocytes was more robust in the lesion core compared to NAWM (Fig. 6b, c; Supp. Figure 6a, b). 3D reconstructions were generated to highlight the prominence and cellular localization of CTSD in lesion core astrocytes (Fig. 6d).

TMEM119 is widely used as a microglial marker; however, it was found to be increased in astrocytes of the lesion core relative to the NAWM and perilesion (Fig. 3b, c). We wondered if TMEM119 was either being expressed or engulfed by astrocytes. Using IF imaging, we labeled chronic active lesions for TMEM119, Iba1, ALDH1L1, and CTSD. As expected, in NAWM, TMEM119 preferentially overlapped with Iba1^+^ microglia/macrophages; however, in the lesion core, TMEM119 appeared clustered within CTSD-labeled lysosomes of ALDH1L1^+^ astrocytes (Fig. 6e, f; Supp. Figure 6a, b). Using 3D reconstructions of labeled astrocytes, we observed prominent lobular TMEM119^+^ staining encapsulated by CTSD^+^ structures (Fig. 6g, h). Notably, TMEM119 often appeared intracellular and granular in both microglia and astrocytes possibly indicating that both cell types were trafficking microglial proteins to either autolysosomes or lysosomes, respectively. To confirm this potential engulfment, additional phagocytic and lysosomal markers were used to label NAWM and the chronic active lesion rim and core. As expected, NAWM and lesion rim Iba1^+^ microglia/macrophages expressed high amounts of CD68 and LAMP1 (Supp. Figure 7a–d). However, in the lesion core, the phagocytic and lysosomal markers CD68 and LAMP1 were prominent in ALDH1L1^+^ astrocytes with Iba1^+^ fragments found encapsulated by LAMP1^+^/CD68^+^ structures (Supp. Figure 7e, f). In contrast, TMEM119 overlapped more prominently with Iba1^+^ microglia/macrophages with little upregulation of CTSD in control tissue, as expected (Fig. 6i, j; Supp. Figure 6c). Similar to this, little upregulation of either CTSD or TMEM119 was observed in ALS tissue (Supp. Figure 3c, f). Interestingly, however, the expression pattern in chronic inactive lesions more closely resembled that seen in the chronic active lesion core where TMEM119 appeared co-localized with lysosomes of ALDH1L1^+^ astrocytes (Fig. 6k, l; Supp. Figure 6d). Given these data, astrocytes may have a critical role in removing dead and dying cell debris within the MS lesion core as another mechanism of fostering a microenvironment conducive to neuronal survival and maintenance.

## Discussion

Astrocytes are ubiquitous throughout the CNS and are critical for the maintenance of homeostasis. In a variety of physiological and pathological settings, astrocytes lie on a spectrum of reactive states, adapting and responding to the microenvironment in which they reside [13]. During pathological conditions, astrocytes exhibit altered morphology and function. This is particularly prominent in chronic active MS lesions, where astrocytes become more reactive and dense in a gradient-like fashion moving from the NAWM, through the perilesion and rim, and particularly in the lesion core (Fig. 1), a lesion area that has been vastly experimentally overshadowed by the reactive lesion rim. This may be due to the thought that astrocytes, a primary component of the chronic active lesion core, have been historically considered pathological bystanders [10, 11]; however, their relevance to MS pathophysiology is becoming increasingly appreciated. Importantly, while astrocytes are a vital source of support to axons, which persist in the lesion core (Fig. 1c, d) [2, 42], astrocyte activation status can also have a critical impact on axonal pathology [43]. Thus, a better understanding of how spatially distinct astrocytes may contribute to the lesion core microenvironment will provide insight into therapeutically targetable pathways.

Spatial protein profiling of astrocytes across four distinct regions (NAWM, perilesion, lesion rim, and lesion core) within chronic active lesions identified the lesion core as having a highly distinct molecular profile. Among the panels analyzed, several proteins of the MAPK pathway exhibited robust expression in the lesion core compared to the NAWM and perilesion (Fig. 3a–c). Specifically, components of the EGFR signaling axis (Fig. 4j), comprising EGFR, pan-Ras, p44/42 MAPK ERK 1/2, and phospho-p90RSK, were the most highly upregulated. Although EGFR signaling can be pro-inflammatory in some contexts, astrocyte-specific deletion of *Egfr* during an immune-mediated murine model of MS led to increased activation of NFκB and downstream expression of target genes, demonstrating that astrocyte-specific EGFR signaling functions to dampen neuroinflammation [44]. Similarly, defective EGFR activity in astrocytes contributed to larger lesions, abnormal glial borders, and impaired functional recovery following spinal cord insult, suggesting that endogenous EGFR activation may be required for proper glial scar formation and protection from secondary tissue damage [45]. Moreover, epidermal growth factor, a primary ligand of EGFR, is known to be reduced in the serum and cerebral spinal fluid of MS patients, suggesting a possible deficit in EGFR signaling during MS [46]. As a result, the functional consequence of elevated EGFR-related proteins in lesion core astrocytes may be to aid in facilitating a tempered astrocytic activation state that contributes to a more quiescent lesion environment.

Astrocytes are heavily integrated with other cell types, engaging in constant intercellular communication. The large bushy processes of astrocytes help to form a wide-reaching cellular network, allowing them to play a central role in regulating the CNS microenvironment. Here, we identified 4-1BB and Tim-3, immune checkpoints that are implicated in modulating autoimmunity and considered targets for immunotherapy [35, 37], as upregulated in lesion core astrocytes (Fig. 5a, f). The upregulation of proteins involved in cell contact-mediated immunomodulation by astrocytes in the chronic active lesion core suggest that these astrocytes influence the inflammatory profile of neighboring cells, including microglia/macrophages. In depth spatial transcriptomic analysis of chronic active lesions demonstrated that while most periplaque white matter microglia expressed the homeostatic marker P2RY12, those in the lesion edge appeared more activated and were predominantly CD68^+^. Interestingly, microglia remaining in the lesion core more closely resembled those in the periplaque, expressing P2RY12 [21], although core myeloid cells vary extensively from lesion to lesion and include both microglia and macrophages. This may indicate that core-associated microglia receive signals, possibly from immunomodulatory astrocytes, to attain a dampened inflammatory profile. Another potent immune checkpoint receptor/ligand pair, PD-1/PD-L1, is known to be expressed in chronic active lesions and its agonism during an inflammatory animal model of MS halted disease progression [30]. While MERTK is highly pleiotropic in function, it has been shown to upregulate PD-L1 expression [39]. Thus, the elevation of both transcript and MERTK protein expression in the lesion core relative to other regions (Fig. 5k–o) highlights yet another pathway that may be employed by astrocytes to reduce the activation of core-associated glia.

While MERTK has been implicated in the regulation of immune checkpoint expression, it is also an important mediator of phagocytosis, particularly efferocytosis, or the engulfment of apoptotic cells [39]. Indeed, MERTK is a phagocytosis receptor shared between astrocytes and microglia and is known to mediate the engulfment of dead and dying cells and synapses. Cell death is prominent during CNS injury and disease, and rapid clearance is necessary to prevent the leakage of intracellular triggers of inflammation [40]. Interestingly, in addition to MERTK, CTSD was also elevated in the lesion core relative to NAWM and the perilesion (Fig. 6), indicating that core-associated astrocytes may preferentially clear apoptotic cell fragments and have the capacity to process the debris via lysosomal proteases. We were initially surprised to find that TMEM119 was prominent in lesion core astrocytes, as it is classically considered a marker of microglia. Further analysis using Iba1 and TMEM119 labeling revealed morphological irregularity of microglia within the core with lobular segments of TMEM119 and Iba1 found co-localized with astrocytic lysosomes (Fig. 6e–h; Supp. Figure 7e, f). This suggests that astrocytes may be engulfing microglial remains to prevent the formation of necrotic cell debris and encourage a more regenerative environment.

Interestingly, the EGFR signaling, immune checkpoint, and phagocytosis markers identified as upregulated in chronic active lesion astrocytes may be specific to chronic focal white matter lesion pathology as nearly all were absent from astrocytes in ALS patient tissue (Supp. Figure 3). However, there was more nuance between chronic active and chronic inactive MS lesion cores with EGFR signaling proteins and indicators of phagocytosis increased in both, while immune checkpoint proteins were found only in chronic active lesion core astrocytes (Figs. 4, 5, 6). Together, this suggests that the role of lesion core astrocytes is active and evolving as it progresses from chronic active to chronic inactive; however, more work is needed to better understand this process.

Spatial protein analyses are powerful tools to uncover the distinct properties of particular cell types while maintaining their anatomical relationship within a given tissue. Here we focused on astrocytes within white matter chronic active lesions in patients with MS. Initially, we found that using GFAP to isolate proteins specifically from astrocytes was problematic in distinguishing separable cells given the dense gliosis within lesions. While ALDH1L1 better separated individual astrocytes, its expression is concentrated in the cell soma (Fig. 2d–e), limiting our ability to analyze proteins expressed by processes and endfeet, portions of the astrocyte that have critical roles in a multitude of astrocytic functions. Nevertheless, the defined protein panels allowed us to parse out a notable and distinctive astrocyte phenotype specific to the lesion core, providing a first glimpse into the microenvironment in which demyelinated axons reside. Based on our findings, astrocytes are active contributors to the preservation of these axons, which is vital to prevent further neurodegeneration and progressive disability in patients with MS.

## Supplementary Information

Below is the link to the electronic supplementary material.

Supplementary file1 (PDF 7552 KB)

## Data Availability

The data that support the findings of this study are available from the corresponding author upon reasonable request.
