# Supplementary material for: Spatial protein profiling reveals active roles for astrocytes in the chronic active lesion core during multiple sclerosis
Source: Acta Neuropathol. 2025 Nov 1;150(1):47. doi: 10.1007/s00401-025-02953-9 (PMC12579715; doi:10.1007/s00401-025-02953-9)
Supplement: Supplementary file 1 — Supplementary file1 (PDF 7552 KB) [file 401_2025_2953_MOESM1_ESM.pdf]

SUPPLEMENTAL FIGURES

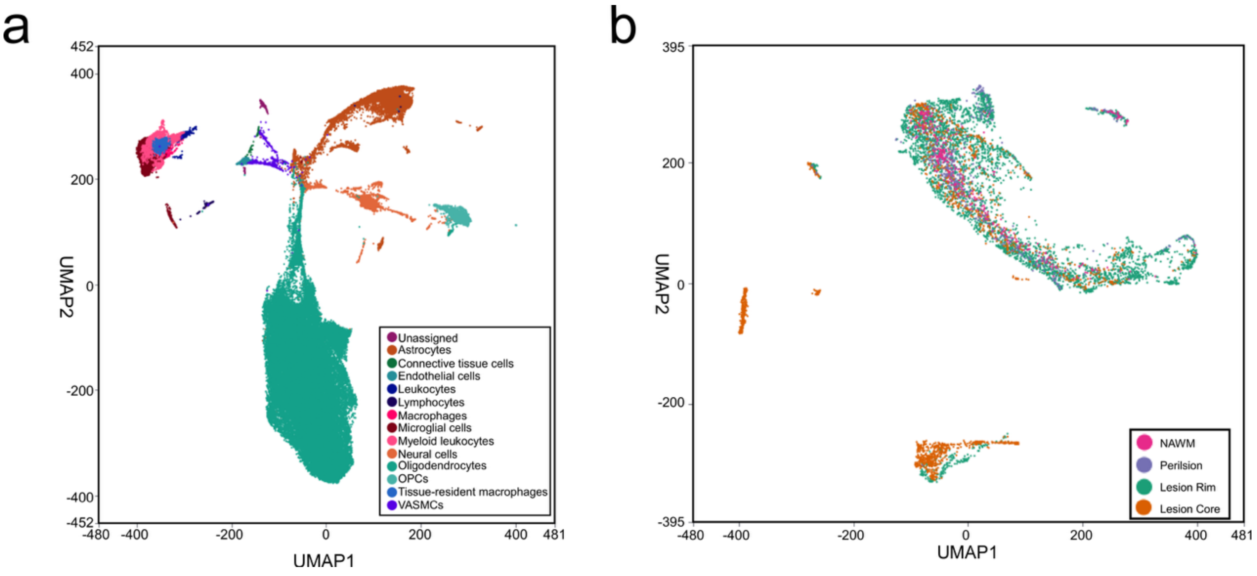

Supplemental Fig. 1 UMAPs of single-nucleus RNA-sequencing data from Abstinta *et al.* [24] (a) Transcript data were subclustered for astrocytes and (b) further subclustered into different regions of chronic active lesions.

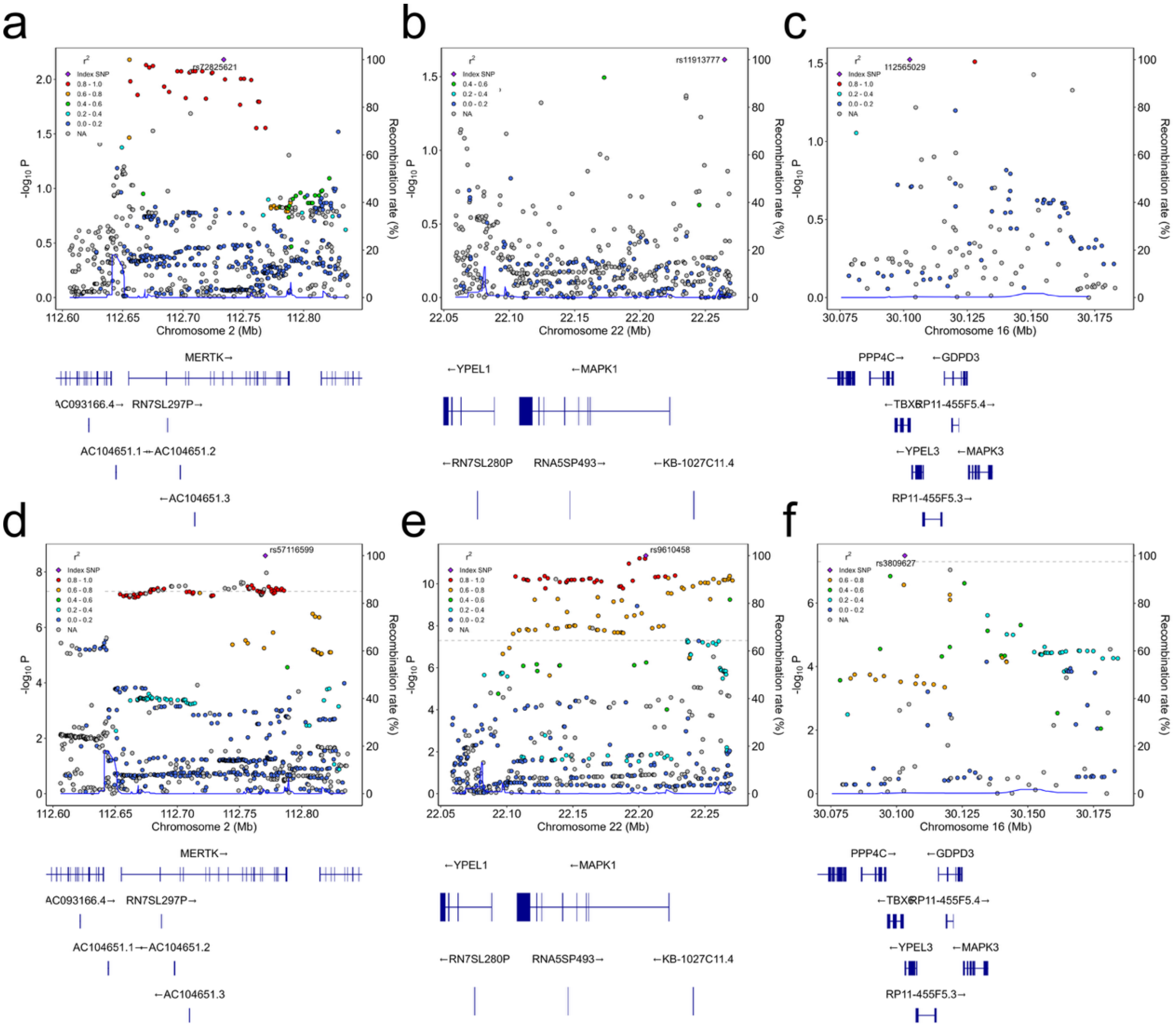

Supplemental Fig. 2 Locus zoom plots of *MERTK*, *MAPK1*, and *MAPK3* gene loci. (a-c) Data derived from the International Multiple Sclerosis Genetics Consortium (MS Severity GWAS) [21] and (d-f) (MS Chip GWAS) [20]. Significant associations were found in *MERTK* (d), *MAPK1* (e), and *MAPK3* (f) in the MS Chip GWAS, associating SNPs in these gene loci with diagnosis of MS.

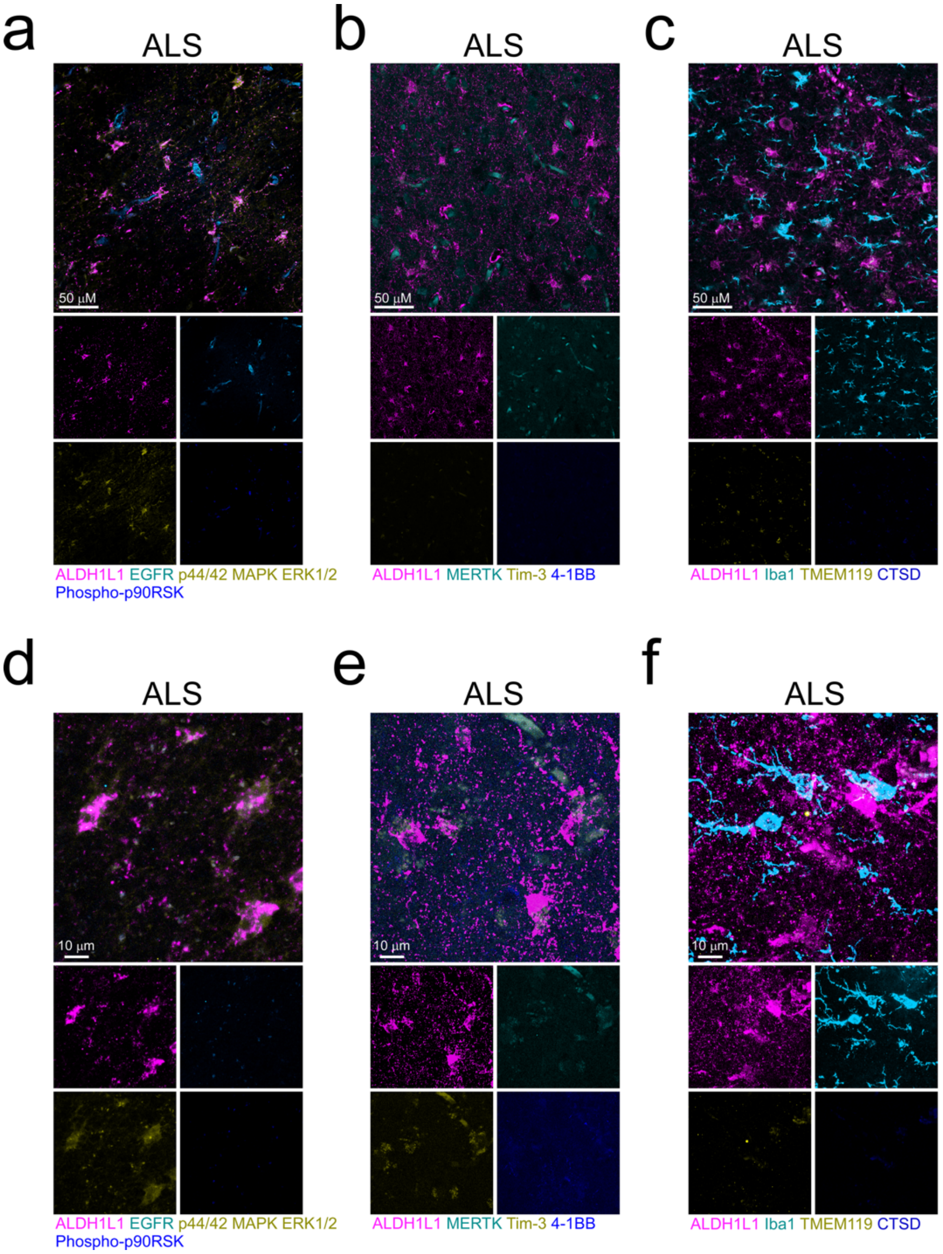

Supplemental Fig. 3 Key astrocytic proteins identified as upregulated in the chronic active MS lesion core compared to NAWM were minimally expressed during ALS. Confocal imaging of ALS tissue labeled for (a) ALDH1L1, EGFR, p44/42 MAPK ERK1/2, and phospho-p90RSK at 20x and (d) 63x magnification, (b) ALDH1L1, MERTK, Tim-3, and 4-1BB at 20x and (e) 63x magnification, and (c) ALDH1L1, TMEM119 and CTSD at 20x and (f) 63x magnification. Scale bars, 50µm (a-c) and 10µm (d-f).

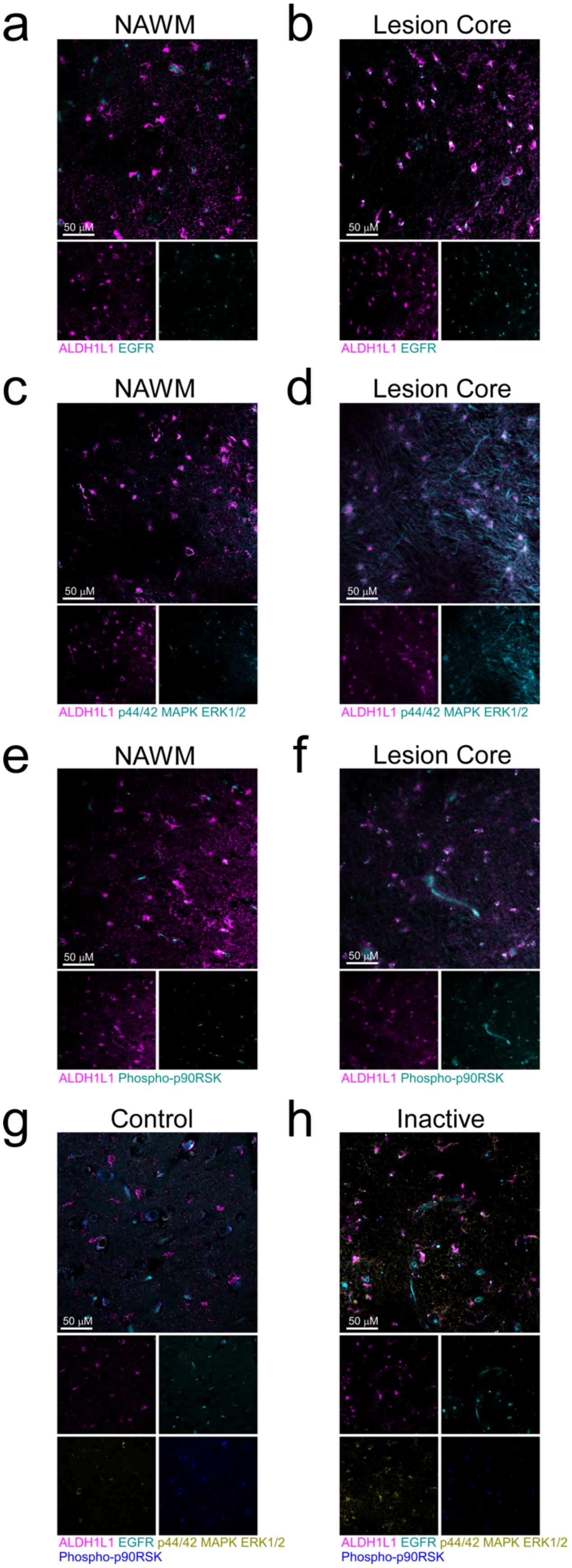
a
NAWM
50 µM
ALDH1L1 EGFR
b
Lesion Core
50 µM
ALDH1L1 EGFR
c
NAWM
50 µM
ALDH1L1 p44/42 MAPK ERK1/2
d
Lesion Core
50 µM
ALDH1L1 p44/42 MAPK ERK1/2
e
NAWM
50 µM
ALDH1L1 Phospho-p90RSK
f
Lesion Core
50 µM
ALDH1L1 Phospho-p90RSK
g
Control
50 µM
ALDH1L1 EGFR p44/42 MAPK ERK1/2
Phospho-p90RSK
h
Inactive
50 µM
ALDH1L1 EGFR p44/42 MAPK ERK1/2
Phospho-p90RSK

Supplemental Fig. 4 MAPK proteins are expressed by astrocytes in the lesion core. Confocal imaging (20x) of (a-b) EGFR, (c-d) p44/42 MAPK ERK1/2, and (e-f) phospho-p90RSK illustrate increased expression within astrocytes in the chronic active lesion core and the (h) chronic inactive lesion core compared to NAWM and (g) control tissue. Scale bars, 50μm.

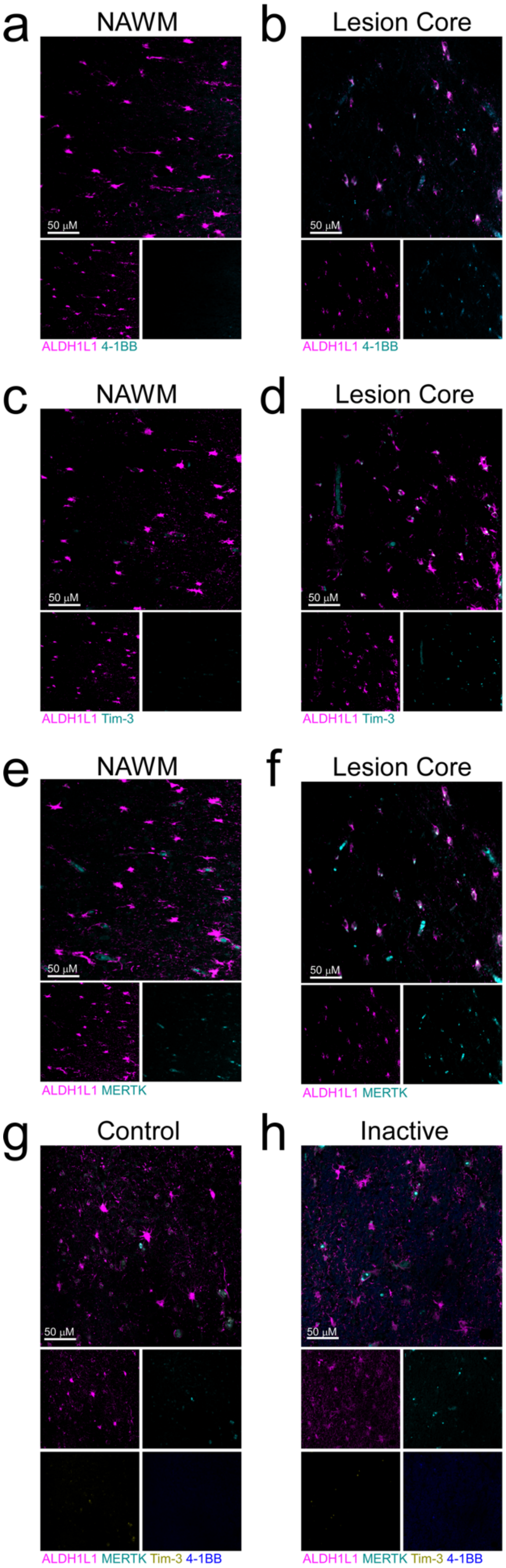
a
NAWM
50 μM
ALDH1L1 4-1BB
b
Lesion Core
50 μM
ALDH1L1 4-1BB
c
NAWM
50 μM
ALDH1L1 Tim-3
d
Lesion Core
50 μM
ALDH1L1 Tim-3
e
NAWM
50 μM
ALDH1L1 MERTK
f
Lesion Core
50 μM
ALDH1L1 MERTK
g
Control
50 μM
ALDH1L1 MERTK Tim-3 4-1BB
h
Inactive
50 μM
ALDH1L1 MERTK Tim-3 4-1BB

Supplemental Fig. 5 Immune checkpoint proteins are expressed by chronic active lesion core astrocytes. Confocal imaging (20x) of (a-b) 4-1BB, (c-d) Tim-3, and (e-f) MERTK illustrate increased expression within astrocytes in the lesion core compared to the NAWM while imaging (20x) in (g) control and (h) chronic inactive lesion core tissue revealed low astrocytic expression. Scale bars, 50μm.

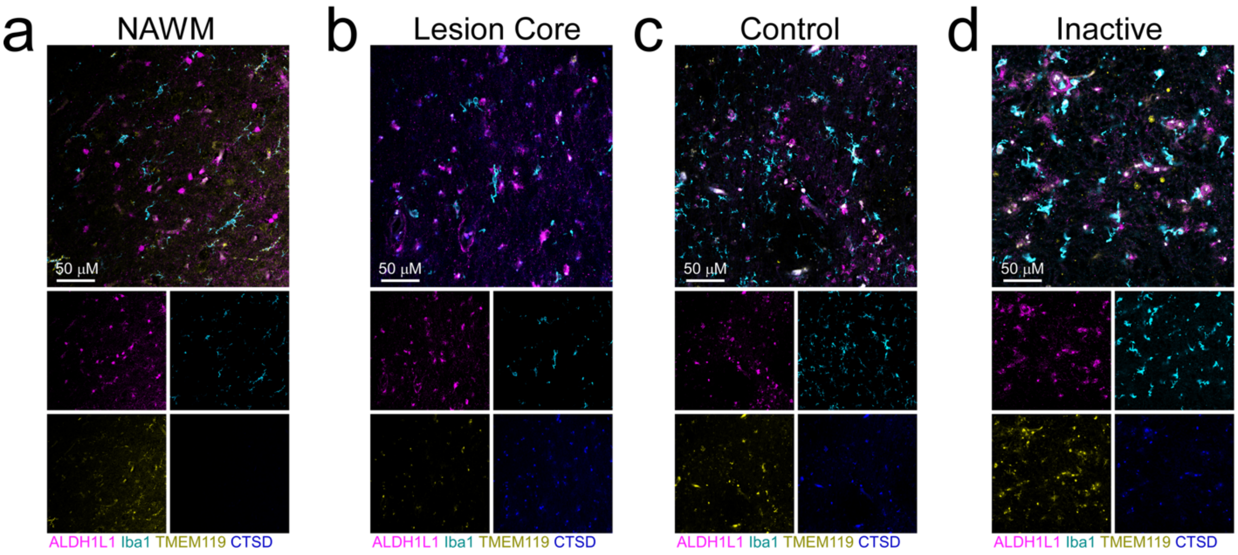

Supplemental Fig. 6 Phagocytosis markers are expressed by astrocytes in the lesion core. Confocal imaging (20x) of ALDH1L1, Iba1, TMEM119, and CTSD illustrate increased co-localization of TMEM119^{+} debris within CTSD^{+} lysosomes in astrocytes in the chronic (a) active and (d) inactive lesion core compared to (a) NAWM and (c) control tissues. Scale bars, 50μm.

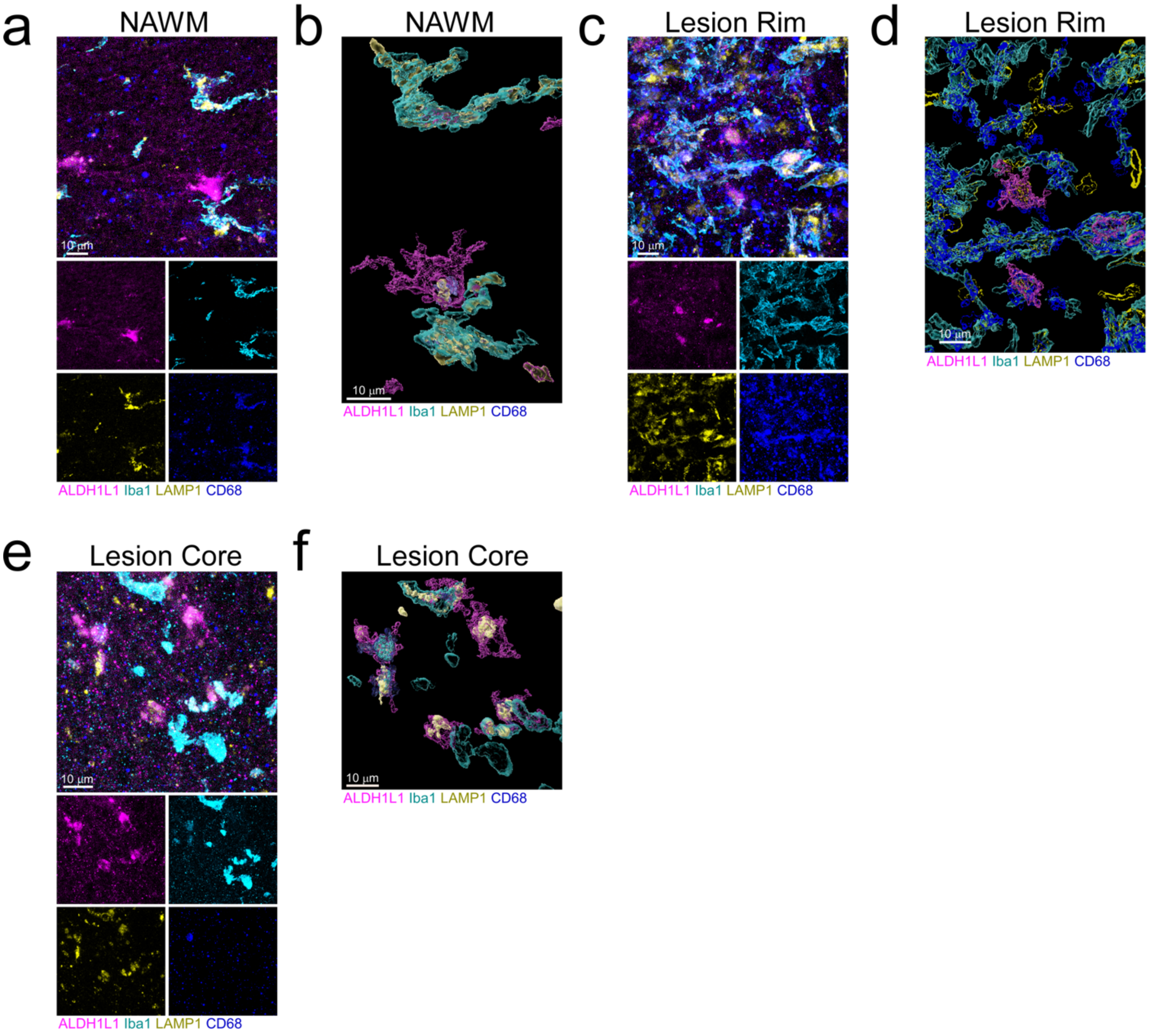

Supplemental Fig. 7 Validation of astrocyte and microglial phagocytic markers in the chronic active lesion. Confocal imaging (63x) (a, c, e) and 3D reconstruction (b, d, f) of ALDH1L1, Iba1, LAMP1, and CD68 illustrate phagocytic (CD68) and lysosomal (LAMP1) markers primarily localized to microglia in (a, b) NAWM and on the (c, d) chronic active lesion rim. However, the (e, f) chronic active lesion core exhibits an increase in CD68 and LAMP1 co-localized with Iba1 within astrocytes. Scale bars, 50µm.
